# Chronic Myeloid Leukemia: A Case of Extreme Thrombocytosis Causing Syncope and Myocardial Infarction

**DOI:** 10.7759/cureus.476

**Published:** 2016-02-02

**Authors:** Rawaa Ebrahem, Brittany Ahmed, Salam Kadhem, Quoc Truong

**Affiliations:** 1 Internal Medicine, University of Kansas School of Medicine-Wichita; 2 Cancer Center of Kansas

**Keywords:** plateletpheresis, apheresis, oncology, syncope, seizure

## Abstract

Chronic myeloid leukemia (CML), a hematologic malignancy characterized by unregulated growth of myelogenous leukocytes, typically presents with symptoms of fatigue, anorexia, and splenomegaly. Laboratory studies often reveal a significant leukocytosis with neutrophilia. A moderate thrombocytosis may be present, but is not usually problematic. The following case discusses a patient who presented with syncope, a convulsive episode, and non ST-segment myocardial infarction secondary to symptomatic thrombocytosis of 2.5 million cells/microL. She was treated with plateletpheresis and subsequently experienced resolution of symptoms. Ultimately, a diagnosis of CML with an atypical presentation of the disease was identified in this patient.

## Introduction

Chronic myeloid leukemia (CML) is a myeloproliferative neoplasm with clonal hyperproliferation of myeloid cells in the bone marrow. The natural course of the disease includes a chronic phase that can progress to an accelerated phase, which can ultimately end in a blast crisis resulting in death [1]. Patients often are diagnosed in the chronic phase, which is somewhat indolent, with clinical findings manifesting as fatigue, decreased appetite, and splenomegaly. Hematologic abnormalities include leukocytosis with neutrophilia and a normal or modestly elevated platelet count. The diagnosis typically is defined by the presence of the BCR/ABL translocation, gene, or protein [2].

We describe a 39-year-old female who presented with syncope, seizure-like activity, and acute non-ST segment elevation myocardial infarction (NSTEMI) secondary to extreme thrombocytosis. This dramatic presentation ultimately led to the diagnosis of chronic myeloid leukemia. Symptomatic thrombocytosis is not a common occurrence in CML, but has been described in case reports with digital ischemia [3,4] and vague neurologic manifestations such as uneasiness and headache [3]. To our knowledge, this is the first case of both neurologic and cardiac complications due to thrombocytosis that has led to the diagnosis of chronic myeloid leukemia. Informed patient consent was obtained verbally for this study.

## Case presentation

A 39-year-old female with a medical history remarkable for a splenectomy secondary to spherocytosis four years prior presented to an emergency department for simple syncopal episodes. She had no prior neurologic or cardiac history. Electrocardiogram was unremarkable, but routine laboratory studies revealed a platelet count of 2.5 million. Due to this abnormality, the patient was referred to a local hematologist and started on a regimen of hydroxyurea and aspirin. Approximately two weeks later, the patient’s son witnessed an episode of shaking movements, apnea, and cyanosis, at which point he called emergency medical services (EMS) for assistance. Upon EMS arrival, the patient was conscious but confused and somnolent. She did, however, report lightheadedness and chest pain prior to the event.

The patient was admitted to the hospital for further evaluation. Laboratory studies again showed a platelet count of 2.5 million, in addition to a leukocytosis of 13,800 cells/microL with 71% neutrophils on the differential. Computed tomography (CT) of the brain was unremarkable and CT of the abdomen and pelvis did not display any lymphadenopathy. She denied any recent weight loss or fevers. Due to the chest discomfort preceding the episode, a troponin was ordered and was elevated; the patient subsequently was transferred to the intensive care unit with a heparin infusion and a cardiology consultation. A transthoracic echocardiogram was unremarkable, as was a CT angiogram of the chest. Electroencephalogram and magnetic resonance imaging of the brain were negative as well.

Hospital Course: Given the negative neurologic and cardiac evaluation, the hematologist suspected that a complication of a myeloproliferative disorder was causing the symptoms. Because her leukocyte count was modestly elevated at 13,800 cells/microL, the hematologist did not believe this would be sufficient enough to cause leukostasis; rather, it was the dramatic thrombocytosis causing the symptoms of vasomotor instability and cardiac ischemia. He elected to perform a mechanical removal of platelets via apheresis. After the reduction in platelet number, troponin level normalized and there were no further occurrences of chest discomfort or neurologic episodes.

A search for the suspected myeloproliferative disorder was conducted, which was initially believed to be essential thrombocytosis. A bone marrow biopsy was obtained and revealed a BCR/ABL translocation, which was confirmed by polymerase chain reaction. This established the diagnosis of chronic myeloid leukemia. Subsequently, she was treated with dasatinib (a tyrosine kinase inhibitor) and discharged to her local hematologist/oncologist for follow-up.

## Discussion

The usual hematologic abnormality of chronic myeloid leukemia is leukocytosis: white blood cell counts often can exceed 100,000/microL [2]. The differential reveals the predominant cells as descending from the neutrophil lineage, present in various stages of development and best visualized on bone marrow biopsy. Our patient demonstrated only a modest leukocytosis of 13,800/microL. Additionally, the splenomegaly that can be present on physical and radiographic findings was not present in our patient who was surgically asplenic.

While leukostasis is a concern in leukemias, it typically presents when leukocyte counts exceed 100,000/microL [5]. Because of our patient’s relatively unremarkable leukocyte count, this led to the suspicion that the grossly elevated number of platelets was responsible for her symptoms. Indeed, thrombocytosis exceeding 600,000/microL can be present in CML [6]; however, platelet counts exceeding 2,000,000/microL are rare and may be related to the patient’s previous splenectomy [7]. In addition, she presented with vasomotor instability and NSTEMI, which have been reported in the literature for essential thrombocytosis but not well described for CML [8]. The mechanism by which thrombocytosis causes symptoms is not fully delineated, but may be related to increased viscosity of circulating blood from transient sludging or aggregation of platelets via a thromboxane-mediated mechanism; this is the rationale for utilizing aspirin as initial medical therapy in thrombocytosis [9].

Two case reports have described digital ischemia due to thrombocytosis in patients with chronic myeloid leukemia [3-4]. One patient was known to have CML and thrombocytosis; she presented with cyanosis in the left hand, in addition to vague neurologic symptoms such as giddiness and headache [3]. A second patient presented with cyanosis of a solitary toe and was diagnosed with CML as a result of a full hematologic investigation, similar to our patient [4].

In both instances, patients were treated initially with medical therapy in the form of hydroxyurea [3], or hydroxyurea in combination with aspirin and allopurinol [4]. Nonetheless, both patients failed medical therapy and continued to remain symptomatic, at which point mechanical removal of platelets via apheresis was successful in alleviating their conditions [3-4]. Plateletpheresis also achieved success in our patient, who demonstrated full resolution of neurologic symptoms and chest pain after reduction in platelet numbers was achieved.

Although symptomatic thrombocytosis is not a common occurrence in chronic myeloid leukemia, it has been described in case reports and successfully treated with platelet apheresis. Once achieved, reduction in platelet numbers resulted in the cessation of symptoms. There has been no evidence to date, however, that plateletpheresis should be used in patients with thrombocytosis who are not experiencing complications [4].

## Conclusions

Symptomatic thrombocytosis caused by chronic myeloid leukemia can have significant consequences that may require urgent hematologic intervention. Plateletpheresis should be considered in these patients. Furthermore, CML should be high on the differential diagnosis for cases of significant thrombocytosis, even when only a modest leukocytosis or neutrophilia is present. These patients should undergo genetic testing for the BCR/ABL gene in order to confirm the diagnosis and tailor therapy. 
